# Clinical, Radiological, and Molecular Findings of Acute Encephalitis in a COVID-19 Patient: A Rare Case Report

**DOI:** 10.7759/cureus.10650

**Published:** 2020-09-25

**Authors:** Saud Bin Abdul Sattar, Muhammad Adnan Haider, Zeeshan Zia, Muhammad Niazi, Qasim Z Iqbal

**Affiliations:** 1 Internal Medicine, Northwell Health-Staten Island University Hospital, New York, USA; 2 Internal Medicine, Allama Iqbal Medical College/Jinnah Hospital, Lahore, PAK

**Keywords:** sars-cov-2, covid 19, acute respiratory distress syndrome [ards], acute encephalitis, seizures

## Abstract

We report a case of encephalitis in a young male patient with severe coronavirus disease 2019 (COVID-19) who initially presented with typical symptoms of fever, dry cough, and shortness of breath but later on developed acute respiratory distress syndrome and required mechanical ventilation. Two days post-extubation, the patient developed new-onset generalized tonic-clonic seizures and confusion. MRI of the brain was done and it showed an abnormal signal in the bilateral medial cortical frontal region. His cerebral spinal fluid (CSF) analysis revealed a characteristic picture of a viral infection with a high white blood cell count and normal glucose and protein levels. After ruling out all common causes of viral encephalitis such as herpes simplex virus (HSV) and based on the review of available literature regarding the neurological manifestations of COVID-19, this case was labeled as acute viral encephalitis secondary to severe acute respiratory syndrome coronavirus 2 (SARS-CoV-2) infection.

## Introduction

In December 2019, a novel coronavirus emerged in Wuhan, China, which resulted in widespread respiratory pneumonia; it was named as severe acute respiratory syndrome coronavirus 2 (SARS-CoV-2), and the disease it causes is called coronavirus disease 2019 (COVID-19) [1]. From its origins in Wuhan, it has now spread across the globe and has been declared a global pandemic and a global health emergency; the COVID-19 pandemic has already claimed thousands of lives worldwide [2,3]. Most of the patients with COVID-19 present with fever, shortness of breath, and cough; however, neurological symptoms such as headache, anosmia, and seizures have also been reported, albeit rarely. Moreover, these patients can further develop neurological manifestations like viral meningitis, encephalitis, Guillain-Barré syndrome, post-infectious brainstem encephalitis, and post-infectious acute disseminated encephalomyelitis [4,5]. Additionally, some studies have claimed that the neuroinvasive potential of SARS-CoV-2 is one of the major underlying causes of respiratory failure in patients with COVID-19 [6].

The first case of acute viral encephalitis secondary to SARS-CoV-2 was reported in February 2020 in China when a patient presented with symptoms of headache, generalized fatigue, and fever, and SARS-CoV-2 RNA was detected in the cerebrospinal fluid (CSF) [7]. This case report, illustrating the findings of encephalitis on brain MRI, is a valuable addition to the growing yet still brief literature on the neurological manifestations of COVID-19.

## Case presentation

In May 2020, during the peak of the COVID-19 pandemic in New York City, a 44-year-old male with no past medical history presented to the emergency department with the chief complaint of low-grade, intermittent, and progressive fever for the past seven days. Fever was associated with non-productive cough and worsening shortness of breath. He denied any features of orthopnea and paroxysmal nocturnal dyspnea. On emergency triage vitals, his oxygen saturation was 89% on pulse oximetry, and hence he was placed on supplemental oxygen via nasal cannula. Initial blood work done in the ER was significant for lymphopenia (auto lymphocyte percent of 3.6%). His chest X-ray showed diffuse bilateral opacities. Based on the presence of the patient in the epicenter of the COVID-19 pandemic and chest X-ray findings, a reverse transcriptase-polymerase chain reaction (RT-PCR) for SARS-CoV-2 was taken from the nasopharyngeal swab and it turned out to be positive. The patient was admitted, isolated (day one), and commenced on hydroxychloroquine, azithromycin, and vitamin C and zinc supplementation (proposed treatment for SARS-CoV-2 at the time of the patient's presentation).

**Figure 1:**
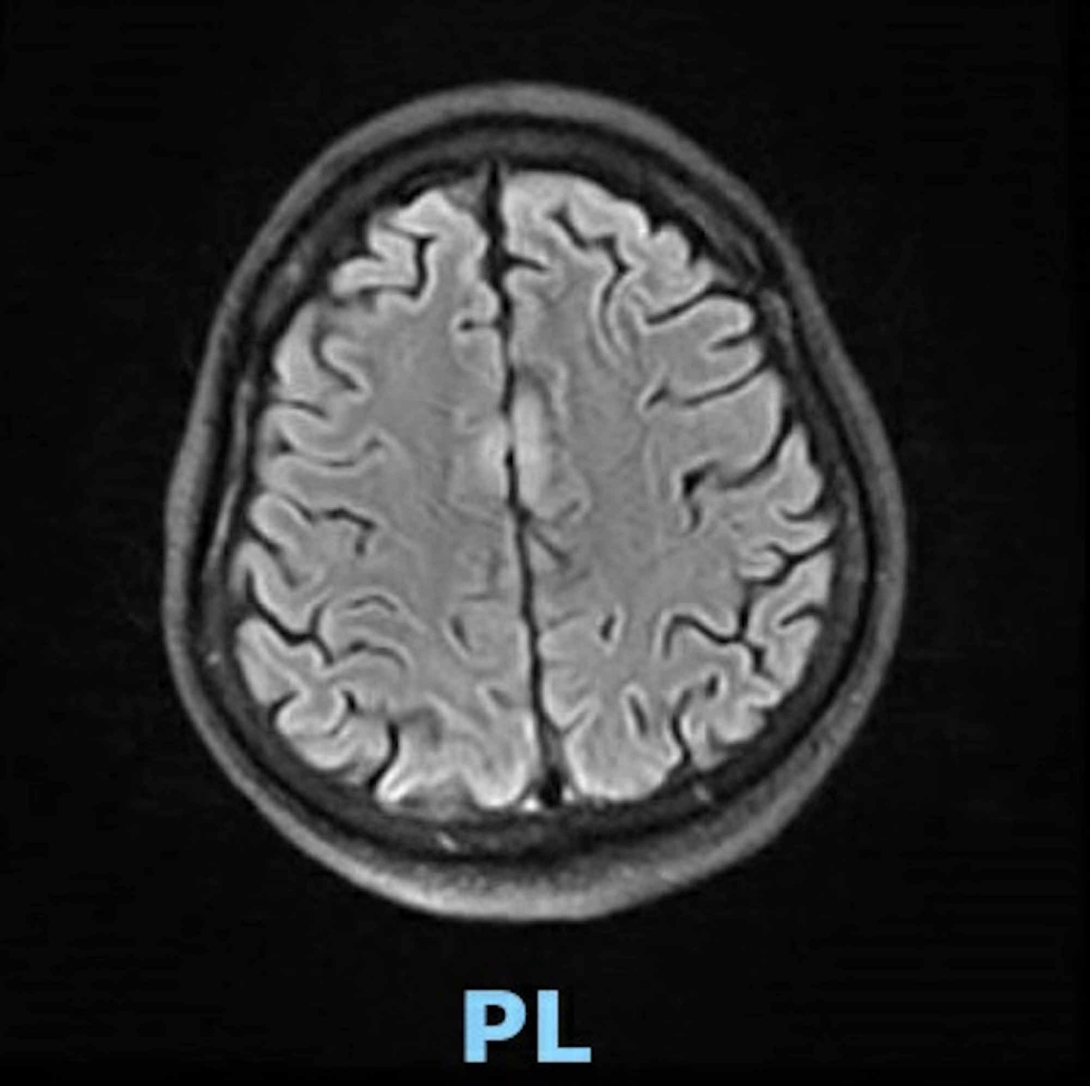
MRI brain showing abnormal medial cortical signals in the bilateral frontal lobes MRI: magnetic resonance imaging

During the hospital stay, the patient’s condition worsened and he developed acute respiratory distress syndrome; he was intubated and put on a ventilator (day seven). He remained intubated for 11 days and was successfully weaned off ventilation on day 18. Two days post-extubation, the nurse witnessed the patient having an episode of generalized tonic-clonic seizures for a minute (day 20). On assessment, he was confused and minimally responsive. Vitals and blood chemistry were within normal limits. The patient was loaded with levetiracetam. An urgent CT head was performed, which showed a few scattered foci of white matter hypo-attenuation and ruled out any structural abnormalities. Lumbar puncture (LP) was performed and was unsuccessful despite multiple attempts (day 20). In order to further investigate the cause of seizures, an MRI of the brain was ordered; but it was delayed to day 25 due to the late availability of the MRI suite. It revealed abnormal medial cortical signals in the bilateral frontal lobes, a sign of encephalitis (Figure 1).

Due to suspicious radiological findings of encephalitis, LP was performed by an interventional radiologist (day 25). Workup for para-infectious/post-infectious encephalitis was done including oligo-clonal bands, myelin basic protein, immunoglobulin G (IgG) synthesis index, angiotensin-converting enzyme (ACE) levels, voltage-gated potassium channels antibodies, para-neoplastic markers, and N-methyl-D-aspartate (NMDA) receptor, which came out as negative. CSF analysis showed normal protein and glucose and mild pleocytosis with a total nucleated cell count of 11 cells/µL (n=0-5 cells/uL) (Table 1). The most common causes of viral encephalitis, herpes simplex virus (HSV) and cytomegalovirus (CMV), bacterial, fungal, and tuberculous were ruled out by microbiological testing and CSF findings. CSF PCR testing for SARS-CoV-2 came out positive. Based on this viral CSF picture in co-relation with clinical and radiological findings, SARS-CoV-2 encephalitis was determined to be the only possible explanation for the patient's condition. His mental status improved gradually as SARS-CoV-2 encephalitis was self-limiting, and he was downgraded from ICU to the medical floor (day 30). There was no relapse of seizure even after stopping the anti-seizures medication and he was subsequently discharged (day 34). The patient was normal without any complaints after many follow-up visits.

**Table 1:** CSF analysis CSF: cerebrospinal fluid

Variables	Results
CSF color	Pinkish
Total nucleated cells counts, CSF	11
CSF eosinophil count	1%
CSF segmented neutrophils count	75%
CSF lymphocytes	21%
CSF monocytes/macrophages	3%
RBC count - spinal fluid	1685
Glucose, CSF	75 mg/dl
Protein, CSF	39 mg/dl

## Discussion

Although patients with COVID-19 usually present with respiratory symptoms of cough, shortness of breath, and fever, symptoms of muscle aches, weakness, and fatigue are also not uncommon in these patients. Moreover, neurological symptoms are also being increasingly recognized and these neurological manifestations vary among different patients. Therefore, every COVID-19 patient should be evaluated for the whole spectrum of neurological symptoms to diagnose and initiate treatment at the earliest [8]. The following three basic mechanisms have been proposed to describe the neuroinvasive potential of SARS-CoV-2.

1. Blood circulation pathway

ACE2 receptors have been identified as functional receptors for SARS-CoV-2, and the severity of symptoms depends on the variable expression and distribution of ACE2 receptors. The presence of ACE2 receptors on neurons, glial cells, and capillary endothelium makes these tissues a target for SARS-CoV-2, and that is why the virus has the potential to invade the central nervous system (CNS) and cause a spectrum of neurological manifestations in COVID-19 patients [9]. The blood-brain barrier is a protective mechanism that hinders the entrance of the virus from the blood circulation into the brain [10]; the SARS-CoV-2, through its S spike protein, interacts with ACE2 receptors of capillary endothelium. The replication and budding then cause damage to the endothelium, resulting in viral entry into the brain milieu [11], and then, the subsequent interaction of the virus with neuronal ACE2 receptors will cause neuronal damage. Inflammatory signals have been detected in the frontal tissue of the brain of a confirmed COVID-19 patient. This strengthens the idea of the neurotrophic potential of the virus and how it can cause encephalitis and seizure [12].

2. Direct infection injury

The other proposed mechanism is the direct entry of the virus into the brain through the cribriform plate; as the virus usually infects and replicates in the nasopharyngeal epithelium, it causes damage to near-by neuronal tissue like olfactory nerves, which can be explained by the symptoms of anosmia in most COVID-19 patients [10].

3. Neuronal pathway

The SARS-CoV-2 virus can also reach the brain via anterograde and retrograde transport through kinesin and dynein proteins of nerves like the vagus nerve [4].

Besides the above-mentioned mechanisms, immune-mediated injury and hypoxic injury of the brain cells are some other possible mechanisms of indirect neuronal injury in COVID-19 patients.

In the absence of other common etiologies of generalized tonic-clonic seizures and the CSF showing a viral picture of encephalitis with negative PCR testing for HSV-1 and HSV-2, our focus shifted towards the SARS-CoV-2 as the underlying cause of encephalitis, as the patient tested positive for SARS-CoV-2 on the nasopharyngeal swab and many neurological manifestations have been reported with SARS-CoV-2 infection. There have been only a few cases of SARS-CoV-2 detection in CSF [7,13], as was in our case. PCR testing for SARS-CoV-2 RNA in CSF is being done in some clinical settings, but it is not a sensitive test. Therefore, we recommend devising more sensitive and specific tests such as CSF inflammatory markers or anti-envelope IgM for the early detection of this virus in the CNS in patients with COVID-19 who present with neurological manifestations [14,15].

## Conclusions

Neurological manifestations of COVID-19 are rare, but in patients who develop neurological symptoms like seizures or confusion, there is a need to detect the presence of this virus in the CNS. However, as PCR testing for SAR-CoV-2 RNA in CSF is not sensitive, we recommend devising sensitive and specific tests for the early detection of this virus in the CNS in patients with COVID-19 who present with neurological manifestations. Furthermore, with a rapidly rising toll of COVID-19 patients developing neurological manifestations, there is a pressing need to understand and diagnose the neurological complications earlier in the course of the disease. In these dire times, physicians should have a very low threshold of suspicion of COVID-19-related neurological manifestations to quickly stratify the patients according to the severity of symptoms and, more importantly, to prevent the long-term neurological sequelae.
